# Determining the cause of inconsistent onset-season trends in the Northern Hemisphere snow cover extent record

**DOI:** 10.1126/sciadv.adv7926

**Published:** 2025-10-31

**Authors:** Aleksandra Elias Chereque, Paul J. Kushner, Lawrence Mudryk, Chris Derksen

**Affiliations:** ^1^ Department of Physics, University of Toronto, 60 St. George St., Toronto, ON M5S 1A7, Canada.; ^2^ Climate Research Division, Environment and Climate Change Canada, 4905 Dufferin St., North York, ON M3H 5T4, Canada.

## Abstract

While seasonal snow cover extent (SCE), an essential climate variable, has broadly declined as a response to global warming, notable inconsistencies remain among long-term satellite-based estimates of SCE change during the Northern Hemisphere snow onset season. SCE datasets from a reanalysis-driven simple snow model serve as benchmarks and allow us to reconcile the trends from one prominent snow cover record with other recent studies. In particular, artificial increasing snow cover trends in the National Oceanic and Atmospheric Administration’s Snow Cover Extent Climate Data Record (CDR) during the onset season are related to changes in snow detection sensitivity. This artificial drift primarily affects September, October, and November snow cover but is detectible through February. Revised trends produced by merging the last decade’s CDR estimates with the offline model datasets reveal decreasing Northern Hemisphere trends in all months but January. This approach shows that offline snow models produce useful benchmarks that can expose biases in observational snow cover datasets with other cross-validation.

## Teaser

With “better glasses” on, satellites detect more snow cover now than 40 years ago, but this optical illusion should be corrected.

## INTRODUCTION

Snow cover undergoes large seasonal variations that shape local ecological conditions (*1*–*3*) and influence the climate system through the surface energy budget [including the snow-albedo feedback; (*4*–*6*)], soil moisture (*7*, *8*), and freshwater release (*9*, *10*). These interactions establish snow as an essential climate variable [ECV; (*11*)] and underscore its sensitivity to climate change. Global warming has been strongly linked to declines in snow cover extent (SCE) through process studies and climate model simulations (*12*, *13*). Hence, far-reaching effects of snow cover changes are expected and continue to be an important avenue for research (*14*).

Across the observational record, composed of both satellite and in situ datasets, expectations of snow cover declines are borne out for spring SCE (*12*, *15*). However, uncertainty remains regarding onset season trends primarily due to the National Oceanic and Atmospheric Administration Snow Cover Extent Climate Data Record [NOAA CDR, (*16*–*18*)], the longest available satellite snow dataset. Despite its widespread use, the NOAA CDR shows increasing SCE during the onset season (+1.5 million km^2^ per decade for October, for example), which contradicts other observational products (*15*, *19*–*23*). Assessments of SCE generally rely on satellite and modeled datasets, given the difficulty of capturing the spatial variability of snow cover with in situ observations. Cloud cover can limit some types of satellite observations, while modeled snow depends on the quality of the forcing data and model accuracy. Cross-validation is therefore a useful tool to combine the strengths of different types of datasets. The NOAA CDR discrepancy increases uncertainty and complicates the characterization of the seasonal cycle of snow cover trends. Within the Global Climate Observing System Framework, guidelines are given for dataset characteristics required to monitor ECVs. For snow cover, the goal stability is below 1% drift in bias per decade. One published study has found a positive trend in the onset-season snow cover duration bias from the NOAA CDR compared to in situ observations from 1992 to 2015, suggesting lack of temporal instability in the NOAA CDR (*22*). These issues also limit community assessments of model performance (*24*), such as in the Coupled Model Intercomparison Project (*25*) and projections of future risks related to SCE changes. Several past studies have strongly suggested that the positive trend in October from the NOAA CDR is spurious and, furthermore, that the variability may be too large in the spring (*15*, *19*, *22*, *23*). For this reason, statistical corrections have been applied before combining products in multi-dataset estimates of snow cover (*12*, *20*).

Here, we explore a hypothesis that has been present in this debate: that the positive trend in October is caused by increased sensitivity to thin snow cover over the course of the satellite record. In practice, this could be the complex result of changes to data volume, instruments, spatial resolution, and data processing algorithms (*16*, *17*). We find evidence of the proposed mechanism by reverse-engineering the SCE and long-term SCE trend for October, and then we expand the study and find that a similar problem appears through most fall and winter months. This approach leads to our method for correcting the NOAA CDR trends. We present a set of monthly snow cover trends, constrained by recent observations, for use in future multi-dataset estimates. This supports the current best practice of using blended, multisource reference datasets for historical snow assessments (*12*, *26*–*30*).

The focus of this work is Northern Hemisphere SCE for the 1980 to 2020 period, which we assess using the NOAA CDR and three reconstructions of historical snow conditions (*31*). The snow reconstructions, produced from reanalysis-driven standalone snow model runs, provide simplified but robust benchmarks for snow cover. These benchmarks capture the variability in the snow caused by surface temperature and precipitation variability. As with other modeled datasets, they are subject to the limitations of the forcing data, such as temporal drifts or spatial discontinuities (*32*, *33*). However, these reconstructions have been evaluated using in situ snow depth observations (*34*) and cross-comparison with a large suite of modern snow datasets (*35*). In both assessments, the reconstructions have been found to provide estimates of Northern Hemisphere snow consistent with the evaluation ensemble.

The snow reconstructions are preferred for the present application because they are free from temporal issues (i.e., step discontinuities and spurious variability) stemming from changes in the data assimilation of snow observations that can be present in the snow output from some reanalysis products [namely, European Centre for Medium Range Weather Forecasts (ECMWF) reanalysis, version 5 (ERA-5) and Japan Meteorological Agency 55-Year Reanalysis [JRA-55, (*22*, *27*, *31*)]. We leverage the daily output frequency of these reconstructions to produce synthetic reconstructions of the NOAA CDR sampling.

## RESULTS

### Reconstructed and observed snow cover variability in October

**Fig. 1.**
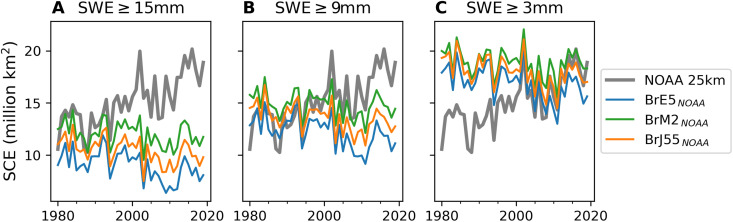
Fixed thresholds in October. SCE (based on land between 40° and 90°N) derived by applying fixed SWE thresholds (as labeled in **A** to **C**) to reconstructed snow cover generated with B-TIM and reanalysis driving data.

We first assess the time series of October SCE from the satellite and offline reconstructed snow cover datasets. Figure 1 confirms that the NOAA CDR over the Northern Hemisphere land (40° to 90°N) shows increasing October snow cover (gray line), opposing the trend from the offline snow cover reconstructions. There are three reconstructed snow datasets, which result from using different reanalysis data to force the Brown Temperature Index Model (B-TIM; see Methods and Materials). They are labeled by the forcing dataset and abbreviated as BrE5 (ERA-5), BrM2 [Modern Era Retrospective Analysis for Research and Applications, version 2 (MERRA-2)], and BrJ55 (JRA-55) (*31*, *36*–*38*). For both the satellite and reconstructed snow datasets, SCE is calculated from the total area of grid cells classified as snow-covered. We classify grid cells from the reconstructed snow datasets as snow-covered when the snow water equivalent (SWE) exceeds a chosen threshold. Temporal subsampling of the reconstructed snow cover datasets establishes a fair comparison to the satellite snow cover (see Methods and Materials). We produced a range of SCE estimates from the reconstructed snow cover datasets, one for each threshold ranging from 0.5 to 18 mm in 0.5-mm increments, although just three thresholds are shown in Fig. 1. For very high (15 mm), high (9 mm), and low (3 mm) thresholds, in Fig. 1, (A to C), trends from all three reconstructed snow cover datasets are consistently negative. This discrepancy is in line with previous studies (*12*, *19*, *20*, *22*). For all datasets, the October snow cover is highly variable from year to year, sometimes differing as much as 50% from one year to the next.

Among the reconstructed snow cover datasets, the interannual variability in the time series is highly correlated, although they are based on different forcing data and/or different thresholds (see the Supplementary Materials for Kendall-tau correlations). The trend discrepancy is the key reason that the NOAA CDR disagrees with the reconstructed snow cover datasets. If the linear trend is removed, the reconstructed snow cover datasets are also strongly correlated with the NOAA CDR (see the Supplementary Materials and table S1). With the linear trend removed, the best agreements result for thresholds between 2 and 4 mm when comparing BrE5, BrM2, and BrJ55 to the NOAA CDR. The four datasets agree on 6 of 10 of the top (bottom) positive (negative) SCE anomaly years (table S2).

**Fig. 2.**
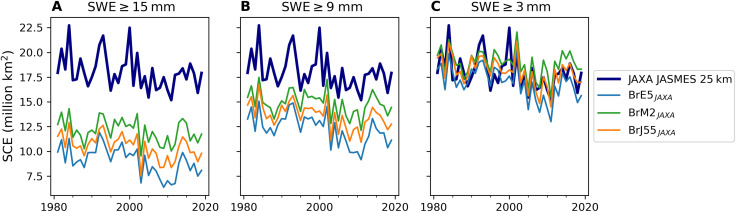
Fixed thresholds in October, with JAXA JASMES. SCE (based on land between 40° and 90°N) derived as Fig. 1 but using JAXA JASMES validity dates.

A key pattern emerging from Fig. 1 is that there is no single fixed SWE threshold that can reproduce the NOAA CDR for October over the whole period. This contradicts the physical expectation that we should be able to relate a realistic map of snow depths to a snow cover estimate through a single fixed threshold. With another satellite-based SCE dataset [JAXA JASMES SCE, derived from objective analysis of AVHRR and MODIS imagery, (*20*)], we can find a fixed threshold that can reproduces the full period (Fig. 2). While limitations inherent to the B-TIM, or any numerical model, mean that there may be drivers of snow variability that are not represented or which drift over time, we see that the first-order effects of temperature and precipitation on snow are captured. In addition, fig. S1 shows trends derived for all the datasets, with shading indicating the range of trends for different thresholds applied to the B-TIM reconstructions. NOAA CDR trends cannot be reproduced throughout the onset season for any threshold, while the B-TIM trends are generally consistent with the JAXA JASMES SCE. BrM2 trends are weaker but consistent with the native MERRA-2 reanalysis, indicating that the weak trends are not related to model complexity. Overall, cross-comparison suggests that B-TIM reconstructions do credibly capture snow cover trends.

In the NOAA CDR case, a decreasing SCE threshold (a gradually less conservative snow-cover classification) appears to reproduce what the satellite has captured. This leads us to exploring the hypothesis mentioned earlier: that an increase in sensitivity to thin snow cover may have artificially caused the increasing October SCE (*19*, *22*). Our thresholding method allows us to explore this in detail. We ask: To reproduce the NOAA CDR, how would the threshold have to vary over time?

**Fig. 3.**
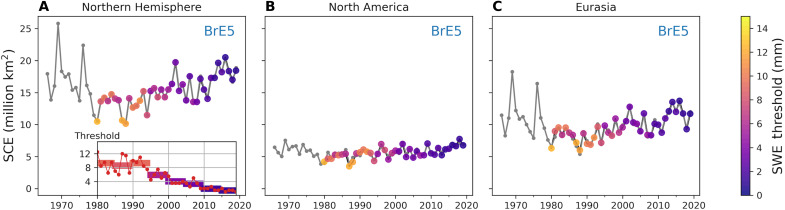
Best-fit threshold values for October. (**A**) SCE (40° to 90°N) generated by tuning the SWE threshold to reproduce NOAA SCE values using BrE5 snow model output. Inset plot shows the yearly best-fit threshold values along with 5-year means for BrE5. (**B** and **C**) The same thresholds as in (A) but summed over North America and Eurasia, respectively.

**Fig. 4.**
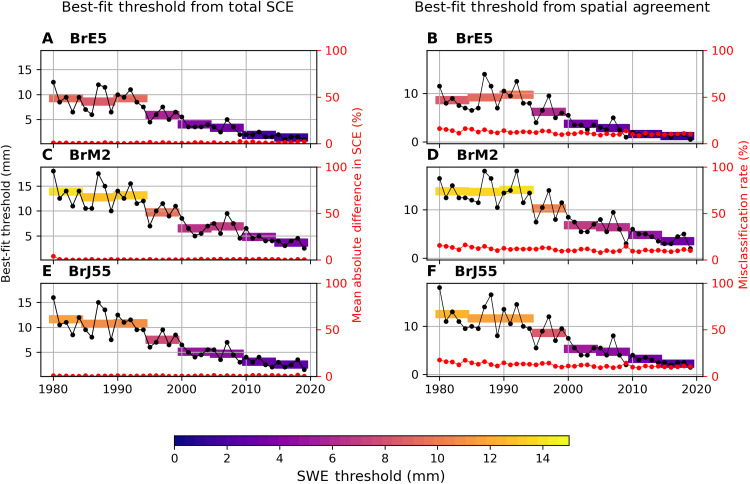
Best-fit thresholds using two methods for October. Yearly best-fit threshold values and 5-year means, calculated by selecting the threshold that minimizes (**A**, **C**, and **E**) total SCE difference or (**B**, **D**, and **F**) the number of misclassified points in each year. Actual misclassification rate shown on right axis as a percentage of all grid cells between 40° and 90°N.

We begin by finding best-fit thresholds for each year and each reconstructed snow cover dataset. The best-fit threshold for each year is chosen by minimizing the absolute difference between the hemispheric SCE from the reconstructed snow cover dataset and the NOAA CDR. This metric rewards global agreement in SCE but does not guarantee that the location of the snow-covered areas agrees. The resulting SCE time series reproduces the NOAA CDR within just more than 1% (root mean square deviation, or RMSD, of 0.17 million km^2^ over the 40-year period compared to a mean of 15.62 million km^2^), with some compensation between the two continents. The RMSD over North America and Eurasia is 0.35 and 0.34 million km^2^ or about 6 and 3%, respectively. Best-fit thresholds found by this method are shown in Fig. 3 for BrE5 using colored markers, with the real SCE value shown along the *y* axis. The inset shows threshold values over time and 5-year averages with the same color map. The Fig. 3A inset is repeated in Fig. 4 alongside the BrM2 (Fig. 4C) and BrJ55 (Fig. 4E) best-fit thresholds. In all cases, the best-fit thresholds needed to reproduce the NOAA CDR decrease over time, as anticipated from Fig. 1. This decrease occurs steadily, without sharp transitions. A similar analysis for JAXA JASMES is shown in fig. S2 with a steady best-fit threshold.

We proceed by using a second metric to assess best-fit thresholds from the perspective of reproducing the spatial distribution of snow cover seen in the NOAA CDR. This second method defines the best-fit threshold for each year as the threshold that minimizes the number of misclassified grid cells (false positives and false negatives) for a given pair of datasets (e.g., BrE5 and NOAA CDR). The misclassification rate is represented as a percentage of the total number of grid cells in Fig. 4. This metric is complementary to the first, rewarding local agreement in the spatial distribution of the snow cover. As before, the best-fit thresholds decrease over time, especially after 1995. Over time, the misclassification rate remains stable (around 10% for this metric) as the threshold evolves.

Together, the results establish that there is a correspondence between satellite snow cover and snow cover derived using thresholding. That is, we can achieve stable spatial agreement and total snow-covered area agreement with the two methods. In both cases, the best-fit thresholds decrease over time, and they agree within 0.5 mm on a 5-year average basis. Consistent results using complementary metrics gives more confidence and reduces the likelihood that our inability to reconcile the satellite and modeled snow cover through a single, fixed threshold is caused by systematic inconsistencies. These inconsistencies could include erroneous model biases in temperature or precipitation that affect where and how much snow falls or step changes in the satellite algorithm (e.g., reclassification of snow grid cells to vegetation or ice).

A decrease in the best-fit threshold over time is consistent with an increasing sensitivity to thin snow in the satellite product. Furthermore, although best-fit thresholds exceeding 9 mm (as seen for early years) are unrealistic, the recent stabilization around 3 to 5 mm is in line with accepted physical relationships between SWE and snow cover (*22*, *39*). This suggests that ongoing improvements to snow detection methodology in the NOAA CDR have brought the NOAA CDR values closer to realistic values. At the same time, this decrease in threshold value has imprinted itself on the record as a positive SCE trend. Although we did not perform in situ comparisons in this work, a recent study that assessed snow cover biases with respect to station measurements similarly found that product-station biases have decreased over time for the NOAA CDR (*22*). Our findings are consistent across the three reconstructed snow cover datasets, indicating that the drift is unlikely to be the result of temporal issues in the temperature and precipitation fields of any individual reanalysis used to force the offline snow model. Similar results appear even as we vary the study region, including considering only land areas between 40° and 60°N (fig. S3) or looking at each continent separately. The magnitude of the trends can differ depending on the forcing data, highlighting the importance of large ensemble studies to cross-validate snow estimates [e.g. (*40*) or fig. S1 shows that BrM2 trends are consistent with output from the more complex snow model in the native MERRA-2 reanalysis]. Although we use multiple forcing datasets, our results are limited to the use of this single snow model. Model-specific deficiencies exist (e.g., for the spring season, as discussed in the next section).

### Full snow-season assessment of snow cover trends

After a focus on October SCE, we extend the same analysis to the rest of the snow-year, beginning with the onset season before looking separately at the mid-winter and spring periods. Although these later periods of the snow season have not drawn as much scrutiny as October, a systematic drift in effective detection sensitivity in the satellite record could plausibly appear in other months.

**Fig. 5.**
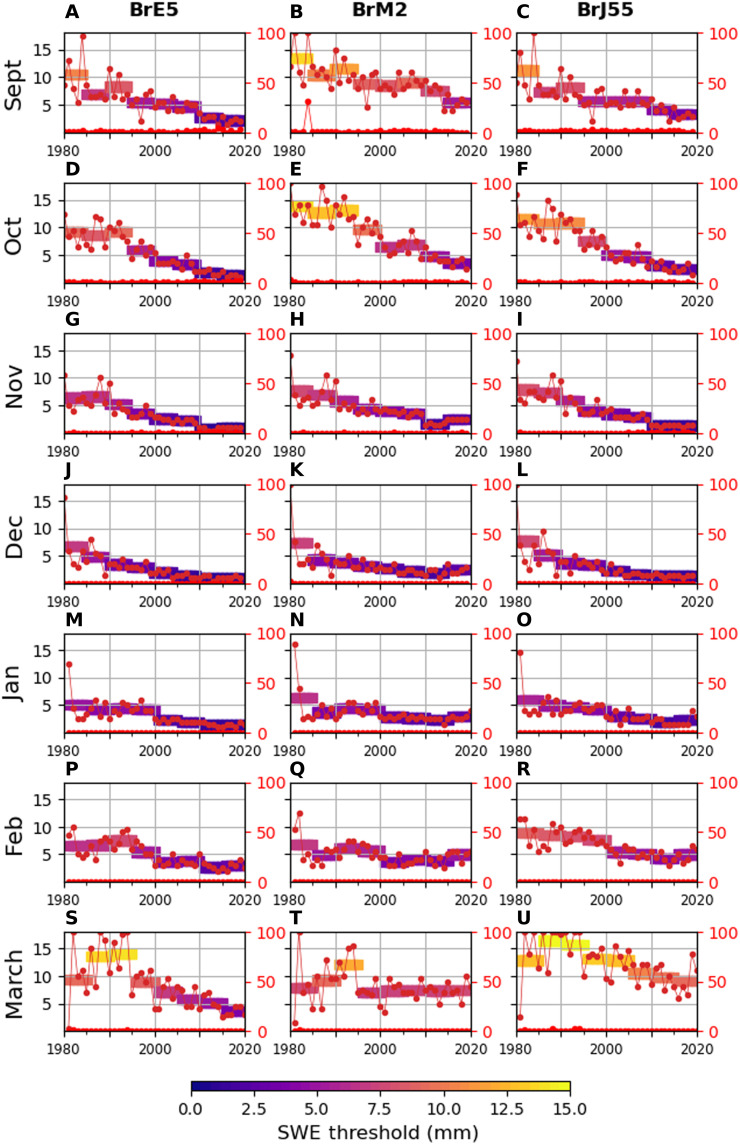
Best-fit thresholds by month. (**A** to **U**) Yearly best-fit threshold values for each month, found by minimizing the total SCE difference between reconstructed snow cover and NOAA CDR SCE. Each column shows the results for a given B-TIM offline dataset (BrE5, BrM2, and BrJ55).

September, October, and November are characterized by the rapid expansion of a relatively thin snow cover over regions without perennial snow. Most of the snow present in September and early October is localized in the Arctic, but the snow line advances quickly to cover large mid-latitude regions through October. Figure 5 (A to C and G to I) shows the evolution of best-fit thresholds for each of the reconstructed snow cover datasets for September and November (October figures are as in Fig. 4 A, C, and E). There are sizeable changes to these best-fit thresholds from the first 5-year period to the last 5-year period (for September, the best-fit thresholds decrease by 9, 8, and 8.5 mm for BrE5, BrM2, and BrJ55, respectively; 9.5, 10, and 10.5 mm for October; and for November, 6, 5.5, and 7.5 mm). The 1980 to 2000 period includes more interannual variability in the best-fit threshold than 2000 to 2020. As before, 5-year averages of the best-fit threshold display consistent decreases over the 40-year record.

In contrast to onset season conditions, the mid-winter snowpack is mostly established. Fewer large fluctuations in snow coverage occur, and large areas have deep snowpacks (SWE > 18 mm, our upper threshold). As a result, the SCE determined by thresholding is less sensitive to threshold variations than in the onset season. December, January, and February display a more moderate best-fit threshold drift, and the best-fit thresholds stabilize earlier in the period (Fig. 5, J to R). Nonetheless, for all months from September to February, the average best-fit thresholds tend toward physically realistic values in the range of 3 to 5 mm in recent years. If we restrict the study region to latitudes between 40° and 60°N, removing Arctic regions to focus on the mid-latitudes, the effective detection threshold displays the same decreasing drift as is seen over the larger region (fig. S4).

The characteristics previously described begin to change in March (Fig. 5, S to U). March is the month typically associated with a maximum of total hemispheric snow mass and the beginning of the melt season. The increasingly important role of snow melt in March and subsequent months is challenging for the B-TIM to capture, and the best-fit thresholds are inconsistent. For BrE5 and BrJ55, the best-fit thresholds do decline over time, but we observe an increase in the 1980s that does not appear for any previous months. In general, the snow depths are overestimated in all B-TIM reconstructions in March, resulting in high best-fit thresholds that exceed physically realistic values. For the remainder of the season, the correspondence between satellite snow cover and snow cover derived using thresholding breaks down (fig. S5). Across a wide range of thresholds, we find that snow-covered area cannot be reconciled with satellite observations for these months. In April, even the highest SWE threshold cannot capture an area as small as what appears in the observations. Later, even the lowest threshold does not capture the snow-covered area in the satellite observations. The B-TIM reconstructions thus overestimate SCE in March and April but underestimate it in May and June relative to the recent NOAA SCE estimates. We attribute this breakdown to limitations of the tool we are using, as the model cannot capture all aspects of the seasonally varying controls on snow cover and snow melt that dominate this period of the snow-year (e.g., increasing role of solar radiation, snow-albedo feedbacks).

Given these findings, we focus the following summary and the final section on the snow onset and mid-winter only. In these months, snow accumulation is the dominant process affecting the snowpack, and the B-TIM reconstructions have been validated using in situ observations and cross-comparison with other data products. We have so far shown evidence that spurious trends appear in monthly snow cover from the NOAA CDR at least between September and February. These trends are consistent with an artificial drift in the NOAA CDR’s effective snow detection threshold, which is not seen in an independent satellite product for which we can find a fixed best-fit threshold. A final important point is that best-fit thresholds have approached realistic values in recent years for all onset and mid-winter months.

### Blended monthly trends of SCE

In this final section, we aim to produce improved estimates of historical monthly NOAA CDR trends by blending BrE5, BrM2, and BrJ55 with appropriately chosen thresholds. We use the NOAA CDR for the threshold calibration, leveraging the most recent observational period for which the NOAA CDR appears to be realistically capturing the snow. Although we have shown that the internal trend makes it unsuitable for trend estimates on its own, it can still provide a useful constraint. We choose the threshold that best reproduces NOAA CDR values for the 2015 to 2020 period for each reconstructed snow cover dataset. Then, we produce snow cover time series extending back to 1980, one for each dataset, and average them before determining the trend. This same method could be used in the future to calibrate threshold choices for other SWE datasets beyond the B-TIM reconstructions shown here.

Table 1 shows mean SCE for 2015 to 2020 for the three regions, based only on the NOAA CDR. Only the Northern Hemisphere total was used for calibrating threshold choices, while the continental values are included to help interpret the resulting trends. The trends are determined from a merged time series with equal contributions from BrE5, BrM2, and BrJ55. Each month’s trends are based on a least-squares linear fit applied to 40 monthly mean values.

**Table 1.** Blended monthly SCE trends. Mean monthly snow-covered area for the Northern Hemisphere (NH), North America, and Eurasia (2015 to 2020 average from NOAA CDR) and estimated trends (1980 to 2020). Trends are derived using the combined estimates from BrE5, BrM2, and BrJ55. Error estimates on the trend values represent the 95% confidence interval on the trend.

	North America		Eurasia		Northern Hemisphere	
Month	Total area (million km^2^)	Trend (million km^2^ per decade)	Total area (million km^2^)	Trend (million km^2^ per decade)	Total area (million km^2^)	Trend (million km^2^ per decade)
Sept.	1.58	**−0.16 ± 0.13**	1.42	−0.18 ± 0.21	3.00	**−0.35 ± 0.23**
Oct.	5.68	**−0.20 ± 0.18**	9.48	**−0.31 ± 0.30**	15.16	**−0.51 ± 0.38**
Nov.	10.74	**−0.24 ± 0.21**	19.46	**−0.57 ± 0.33**	30.20	**−0.81 ± 0.36**
Dec.	13.19	−0.11 ± 0.13	24.47	**−0.29 ± 0.24**	37.67	**−0.40 ± 0.27**
Jan.	13.78	−0.06 ± 0.10	26.03	−0.16 ± 0.26	39.81	−0.22 ± 0.27
Feb.	13.5	0.00 ± 0.14	25.29	**−0.34 ± 0.32**	38.79	**−0.34 ± 0.24**
March	12.36	−0.03 ± 0.17	21.85	**−0.60 ± 0.38**	34.21	**−0.63 ± 0.41**

There are statistically significant trends (at the 95% confidence level) in the Northern Hemisphere SCE for all months but January. All significant trends are negative, and the greatest magnitude of the trend occurs in November, with −0.81 million km^2^ per decade. Most of this snow cover loss is over Eurasia; statistically significant trends in Eurasia are found in all months except September and December, with the greatest magnitudes occurring in November (−0.57 million km^2^ per decade) and March (−0.60 million km^2^ per decade). The statistically significant trends in North America occur only in the onset season (September to November) but represent substantial fractions of the total snow-covered land in that region. Compared to estimates based on the JAXA JASMES snow cover, our trends in the onset season are somewhat stronger, with mid-winter trends in closer agreement [see fig. S1 and table S4, (*20*)].

## DISCUSSION

We have used a range of SWE thresholds to produce reanalysis-driven reconstructions of SCE with the aim of explaining discrepancies in historical snow cover from the NOAA CDR, including apparent increases during the snow onset season. The relationship between reconstructed SWE and snow extent has a physical basis but is model and spatial resolution dependent. However, the crucial assumption is that this relationship should not vary in time. For example, when we compare the offline reconstructed snow cover datasets to the JAXA JASMES snow extent product, a fixed threshold can consistently reproduce the full-time series of SCE (figs. S1 and S2). In contrast, there is no fixed SWE threshold that can accurately reproduce the multidecadal time series of SCE from the NOAA CDR. Instead, the optimal SWE threshold decreases over time. This is direct evidence of spurious positive trends in the NOAA CDR caused by increased sensitivity to thin snow over time. These effects are most prominent in September, October, and November but remain present through the winter. The effective threshold value that relates the reconstructed snow cover datasets to the NOAA CDR has approached physically realistic values in recent years and exhibits less year-to-year variability. This may be due to the increased quantity and quality of information available to the snow analysts who produce the snow charts, or changes to the detection algorithm (*16*, *17*).

The threshold evolution, which we argue reflects the internal trend in the satellite dataset, is most dramatic in the snow onset season. This suggests that rapid changes in snow cover worsen the underlying mechanism that causes underestimates of snow cover in the early satellite record. Gap-filling techniques that rely on previous observation days and previous weeks’ snow maps could be such a mechanism and one which would have weakened in recent decades with more and better data. This mechanism could also suppress the artificial trend in the winter and spring, when the snow cover is retreating, offering a potential answer to the prevailing question about differing behavior in fall and spring [e.g., (*19*)], although we are limited to the onset season.

This analysis identified and explored the large discrepancies between the NOAA CDR and three modeled snow reconstructions, which were mutually consistent despite different meteorological forcing. Supported by comparisons with another observational dataset, we find that the B-TIM reconstructions credibly capture the SCE variability. The reanalysis temperature and precipitation variables have some drift (*32*, *33*). In general, reanalysis systems assimilate a varying number of observations over time, which can affect trends. Although we guard against this effect by using multiple forcing datasets, we are limited using a single snow model that simplifies processes such as forest cover and snow-albedo feedbacks. While the B-TIM, with its two meteorological drivers, benchmarks well against more complete models, it could be beneficial to generate similar reconstructions with other offline models, bearing in mind the need to monitor for errors as more input data are introduced.

Considering the evidence of spurious trends throughout the NOAA CDR, we calculated stand-in snow cover trends using a blend of the reconstructed snow cover datasets. We calibrated thresholds for each reconstruction using NOAA CDR values from 2015 to 2020, the recent period over which the SWE-to-SCE relationship is characterized by physically reasonable detection thresholds. The resulting trends represent changes to SCE over 1980 to 2020 for land areas between 40° and 90°N as they would be captured by a NOAA CDR sampling the modeled snow field. We found statistically significant snow cover losses in all months but January, with the strongest decreases in November. This seasonality in the trends is consistent with other multiproduct estimates (*12*). These methods can be easily applied to produce model-derived trends for additional modeled snow datasets, such as Crocus snow model output, with calibration to the benchmark SCE values in Table 1. There is also potential to correct the NOAA CDR by reprocessing the full record of snow cover maps with modeled daily snow cover estimates such as those provided by the B-TIM products.

Future work remains to be done to extend this analysis throughout the full snow season. This study was limited to the months between September and March because the B-TIM does not reliably capture the rapid snow melt during boreal spring. This may require an albedo parameterization or a more detailed surface energy budget in the snow model used, although there are challenges with this approach. Nonetheless, it would be valuable to have a tool to robustly assess trends for all months, including the melt season.

## MATERIALS AND METHODS

### Snow model (B-TIM)

The reconstructed snow cover datasets used in this study are derived from modeled snow produced with the B-TIM. The version of the model used for this study, v1.0.0, is fully documented and available online (*31*, *41*), although we give a high-level summary below.

The B-TIM model simulates snow accumulation starting from zero snow at each grid cell beginning on 1 August and ending on 31 July of each snow-year. The snowpack evolves according to parameterized relationships representing the following physical processes: melt from rain-on-snow, melt when air temperatures exceed −1°C, warm and cold settling processes that increase the density of the snowpack while retaining the total amount of water, and snow accumulation from new snowfall, determined as 80% of the precipitation that occurs below 0°C. All processes parameterizations depend only on air temperature, existing snow depth, and precipitation, so the model only requires temperature and precipitation. Here, we run the B-TIM with hourly or 3-hourly data from the ERA-5 (*42*), the NASA MERRA-2 (*33*, *43*), and the JRA-55 (*44*).

Modeled SWE and snow density estimates are produced at the spatial resolution of the driving data and at daily frequency. The B-TIM reconstructions described here have been benchmarked against in situ observations of SWE and have been shown to rank in the upper 50% among an ensemble of complex models (*31*, *34*, *35*).

### Thresholding method

Daily B-TIM SWE is converted to binary maps of daily snow cover, SC through a fixed SWE threshold, *SWE*_thresh_, assuming step behavior of the form SC = H(SWE − *SWE*_thresh_) where H is the Heaviside step function. This definition allows us to vary *SWE*_thresh_ to vary the number of grid cells classified as snow-covered. Daily SCE time series are then produced by integrating the areas of snow-covered grid cells for each of the snow cover datasets. We repeat this process for a range of thresholds (0.5 to 18 mm with 0.5-mm increments). The SCE monotonically decreases as the threshold increases, with higher values of *SWE*_thresh_ producing more conservative estimates of snow cover.

### Regions

The two domains we consider, North America and Eurasia, include land between 40° and 90°N unless otherwise specified and do not include Greenland. North America is defined as the region from 170° to 25°W, and Eurasia is defined as the region between 25°W and 190°E.

### Sampling based on NOAA CDR

The NOAA CDR is a record of weekly snow maps. Each map is based on an analysis of satellite observations (and model output, since 2003) collected for over 1 week. The resulting snow cover map is indexed by the last day of that week, the “validity date.” Notably, satellite data sources have not been static over the lifetime of the product, and the snow detection methodology has changed over time. Before 1999, data from the last day the land surface were observed at each grid cell (~190.5-km resolution) was combined with the previous week’s map, and snow boundaries were drawn by trained NOAA meteorologists (*45*). Using this approach, weekly SCE maps were heavily weighted toward the end of the mapping week but could contain some information from earlier in the week. Since 1999, the record contains information from the daily Interactive Multisensor Snow and Ice Mapping System (IMS) binary snow cover product (24 km), replacing the hand-drawn maps. Data from the overlap period of June 1997 to May 1999 were used to calibrate the two methods. It was found that Monday IMS snow cover resulted in the best match with the older method and that a snow-covered grid cell classified with the older method on the coarser grid corresponded to scenes where at least 42% of the grid cells at the new resolution were classified as snow-covered (*16*). Data sources for IMS have continued to evolve, with more recent sources achieving exceptional resolution (down to 1 km) compared to the original SCE maps. Examples of changes to the data sources include the addition of surface analysis (e.g., from SNODAS after 2003), additional instruments (e.g. MODIS observations begin in 2004), and replacements of data sources by next generation instruments [AMSR-E is replaced by AMSR-2 after 2011, (*46*)].

To replicate the NOAA CDR sampling method as closely as possible, we subset the reconstructed snow cover datasets by extracting only data on the NOAA CDR validity dates. This subselection is then treated as representative of the snow conditions during the preceding week. Monthly averages are generated using this subset of validity dates, both for the reconstructed snow cover datasets and the weekly NOAA CDR. All mapping weeks with any days falling in a month *m* contribute to the monthly mean of *m*. For example, if 2 October is a validity date in the NOAA CDR for a given year, the 2 October snow cover map contributes to the calculation of the September and October monthly means: 5 days in September and 2 in October. This reproduces the method used to generate monthly values in the NOAA CDR published at https://climate.rutgers.edu/snowcover/ (last access January 2025).

We apply this method to reconstructed SCE datasets and the weekly NOAA CDR for all 40 years, August 1980 to July 2020. This results in 40 monthly SCE values for each month (for the B-TIM reconstructions, this is repeated for each threshold).

### JAXA JASMES SCE data processing

Using the 0.05° weekly JAXA JASMES snow cover dataset, we have regridded all snow cover maps to the 25-km EASE-Grid 2.0, matching the NOAA CDR. This is done by finding the mode of the surface flag from all grid cells within a target grid cell, collapsing the four flags indicating high and low probability of wet and dry into a single snow-covered flag value. The resulting dataset values indicate grid cells with snow cover, land (no-snow), clouds, or no-data. Wherever there are clouds, if both the previous and following week indicate that a given grid cell is snow covered, we fill the grid cell with snow. Cloud cover ranges from 0 to 20% of the land north of 40°.

Monthly values are derived similarly to NOAA CDR. The B-TIM reconstructions are sampled to the JAXA JASMES validity dates before averaging, as in Fig. 2.

### Statistical analysis

All linear trends are computed using a least-squares fit to the time series of monthly mean values. The significance of each calculated trend is tested using a two-sided Student’s *t* test and a significance level of 0.05. To calculate the 95% confidence interval on the trend, as shown in Table 1, we multiply the SE of the slope from the linear regression with the *t* value from the Student’s *t* distribution corresponding to this confidence level, using degrees of freedom equal to the number of years minus two (*dof* = 38 for our 40-year records).

When discussing detrended time series (e.g., Kendall-tau analysis), we refer to time series *Y*_detrended_ = *Y* – (*mX* – *b*) where *m* and *b* are the two fitted parameters from a least-squares fit to a line with slope *m* and *y*-intercept *b*. If (*X*, *Y*) are ordered pairs of data points, corresponding to a year and an SCE value, we have (*X*,*Y*_detrended_) after detrending.

## Supplementary Materials

### This PDF file includes:

Supplementary Text Figs. S1 to S5 Tables S1 to S4 References
