# Supplementary material for: Determining the cause of inconsistent onset-season trends in the Northern Hemisphere snow cover extent record
Source: Sci Adv. 2025 Oct 31;11(44):eadv7926. doi: 10.1126/sciadv.adv7926 (PMC12577703; doi:10.1126/sciadv.adv7926)
Supplement: Supplementary file 1 — Supplementary Text Figs. S1 to S5 Tables S1 to S4 References [file sciadv.adv7926_sm.pdf]

Science Advances
AAAS

Supplementary Materials for

Determining the cause of inconsistent onset-season trends in the Northern Hemisphere snow cover extent record

Aleksandra Elias Chereque *et al.*

Corresponding author: Aleksandra Elias Chereque, aleksandra.eliaschereque@mail.utoronto.ca

Sci. Adv. **11**, eadv7926 (2025)
DOI: 10.1126/sciadv.adv7926

This PDF file includes:

Supplementary Text
Figs. S1 to S5
Tables S1 to S4
References

Supplementary Text

Comparing coherence of SCE time series

Snow cover records are sometimes used to partition the historical record into high and low snow cover anomaly years, most often removing a running mean or long-term trend from the record. In light of this, we assess the similarity between each reconstructed snow cover dataset and the NOAA CDR using the Kendall-tau correlation on detrended data (*47*). This correlation coefficient quantifies the extent to which the ordered list of years, ranked by snow cover anomaly, matches. Therefore, this comparison captures how sensitive the published results are to changes in baseline dataset if they depend on such rankings.

The Kendall-tau correlation statistic ranges from -1 to +1 and is maximized when two rankings of the same items are identical (i.e. the same years are ranked first, second, third, etc.). The statistic is equal to -1 when the rankings are inverted. The statistic tends to zero when the orderings are very different.

We find that the temporal variations in SCE are highly coherent across all datasets. This is quantified in Table S1 with Kendall-tau tests applied to detrended October SCE time series. All results are significant at the $p<0.025$ level with dof=40.

1. Same forcing: For the same B-TIM reconstruction, SCE produced with different thresholds is highly correlated. The strength of the correlation decreases when there is a greater difference between the thresholds being compared, but tau values exceed 0.5 for all possible pairs of this type. (Not all pairs are shown in Table S1.)
2. Cross-pairs: For the same threshold, SCE from different B-TIM reconstructions is also highly correlated, with values exceeding 0.7. There is neither a strong threshold dependence nor dataset dependence to this result.
3. NOAA CDR: The correlations between the B-TIM reconstructions and the NOAA CDR are somewhat weaker than for the same-forcing pairs or cross-pairs. The tau values decrease with increasing threshold, and are greatest for threshold values between 2 mm and 4 mm.

As another measure of similarity, we can compare the lists of 10 years with highest and lowest snow cover (after detrending) from each dataset. 8 of 10 “high snow years” appear in common across the B-TIM offline snow datasets, 6 of which also coincide with the high snow cover years from the NOAA CDR dataset. For the lowest snow cover years, the B-TIM offline snow datasets identify 7 of the same years, 6 of which also appear based on NOAA CDR. This is consistent with the Kendall-tau results in Table S1 in that tau values in the NOAA CDR comparison are lower overall than in the cross-pairs comparison.

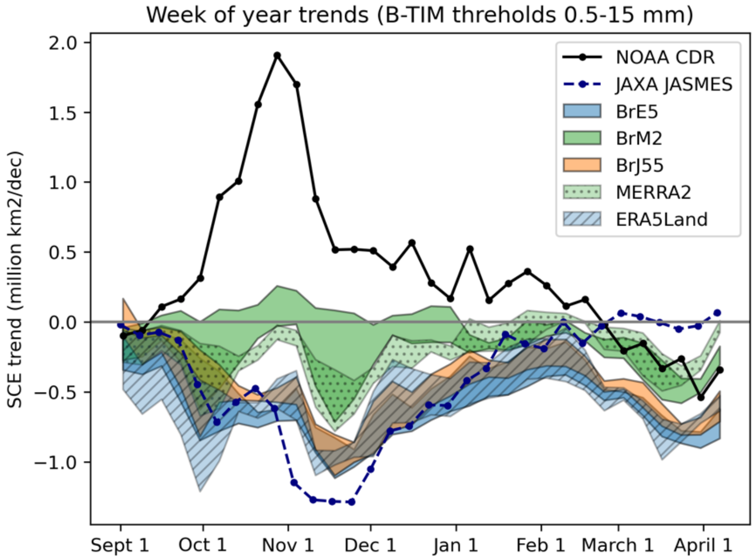

Fig. S1

Weekly trends from snow datasets. All datasets except JAXA JASMES are first subsampled to the NOAA CDR validity dates. Weekly SCE values are grouped by week of year and linear trends are calculated across the 40-year period. MERRA2 and ERA5Land are reanalysis datasets which do not directly assimilate snow observations, so they are comparable to the B-TIM datasets. This figure illustrates the result that NOAA trends in the onset season are distinct from all modelled datasets across all fixed thresholds. The models are consistent with the range of estimates from reanalysis and another independent satellite dataset.

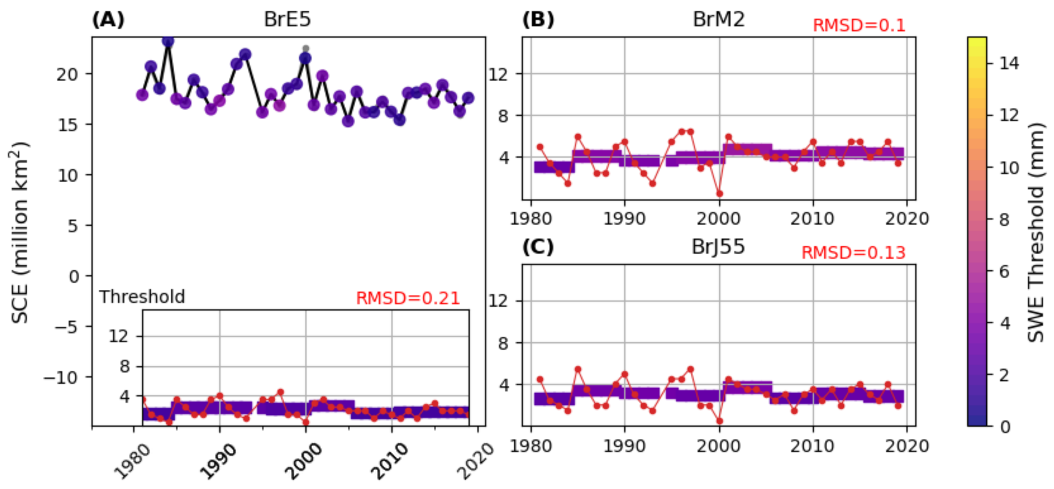

Fig. S2

Best-Fit threshold values to JAXA JASMES for October. Same analysis as Fig. 3 but using JAXA JASMES SCE as the target instead of NOAA CDR. Best-fit thresholds are physically realistic and stable over time.

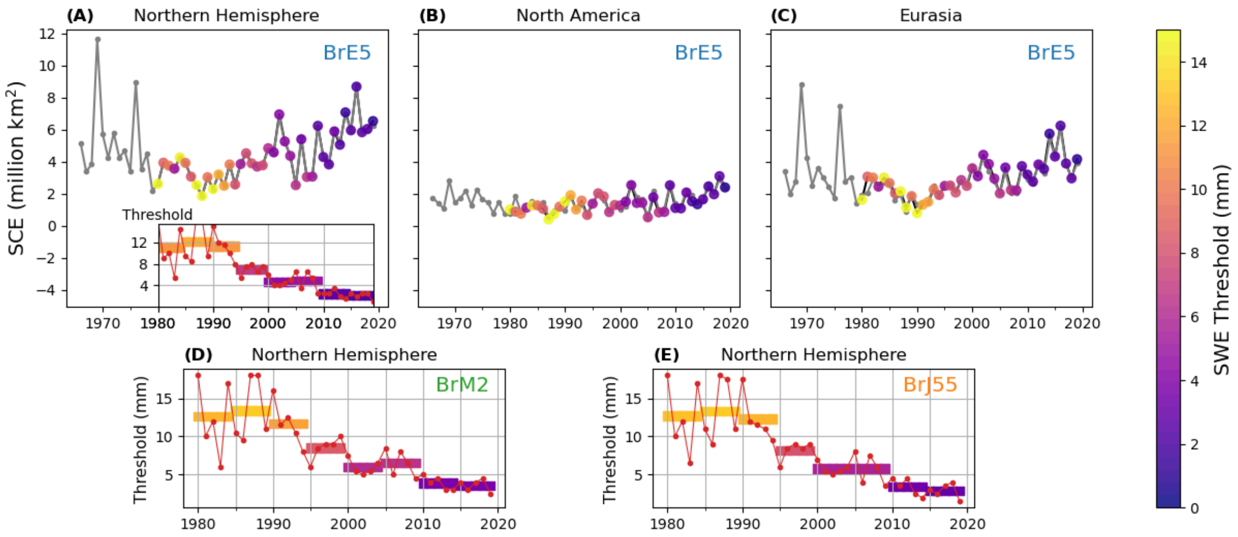

Fig. S3

Best-fit threshold values by region for October. As in Fig. 3 but based only on data from 40°-60° N. Panels D, E show the best-fit thresholds for this region using BrM2 and BrJ55, respectively.

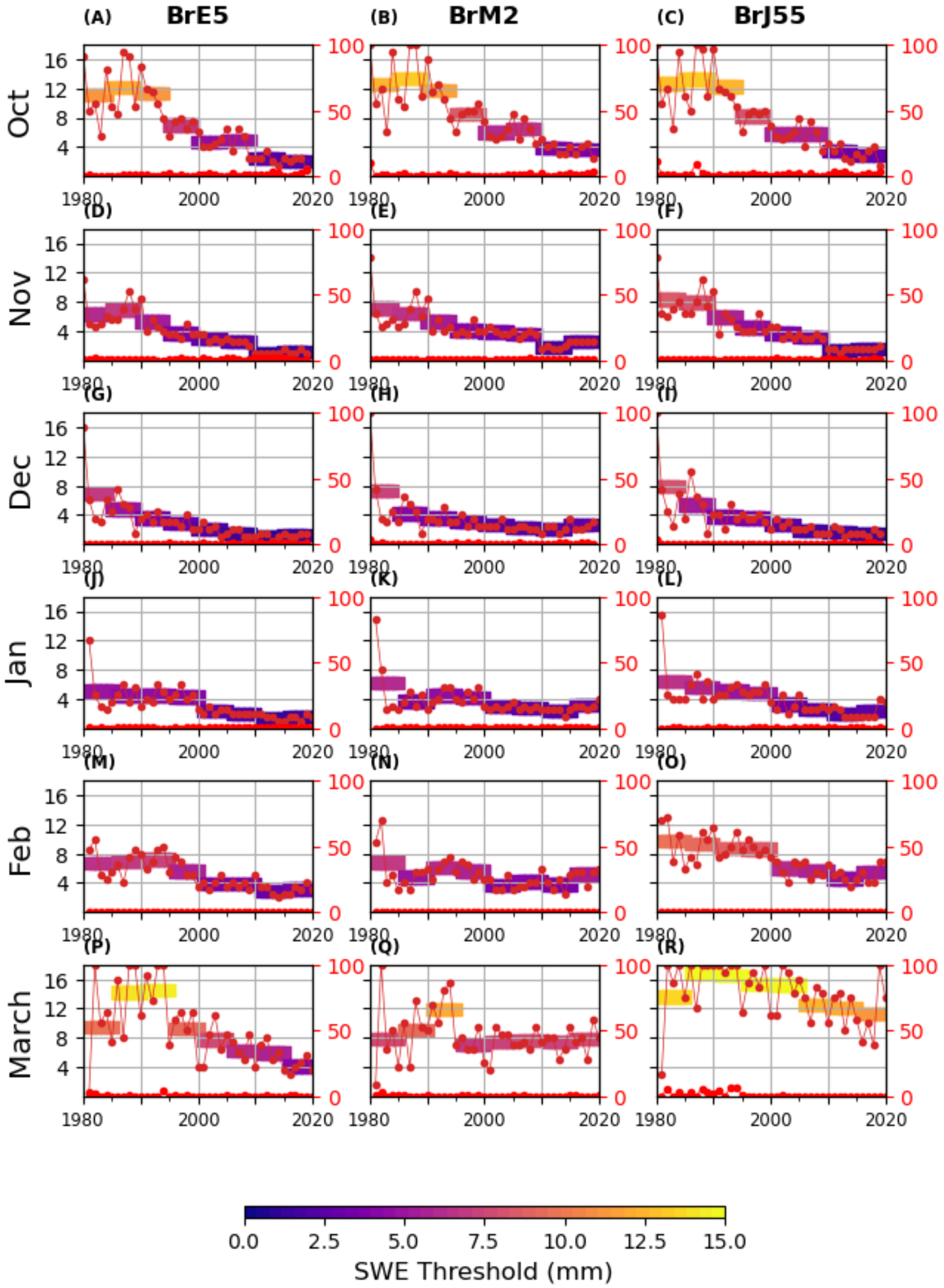

(A) BrE5
(B) BrM2
(C) BrJ55
(D)
(E)
(F)
(G)
(H)
(I)
(J)
(K)
(L)
(M)
(N)
(O)
(P)
(Q)
(R)
Oct
Nov
Dec
Jan
Feb
March
SWE Threshold (mm)

Fig. S4
Best-fit threshold values by area agreement for 40°-60°N only. As in Fig. 4, but for midlatitudes only. We exclude September because in this region, snow does not significantly appear until later in the season. RMSD is shown in the upper right corner in units of million km^2.

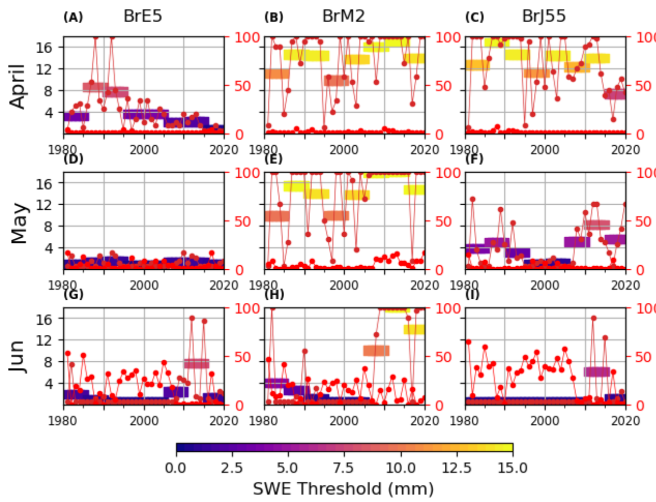

Fig. S5

April-June best-fit threshold by area agreement. RMSD is shown in the upper right corner in units of million km^2.

Thresholds (mm)					
		**BrE5-BrE5**	**BrM2-BrM2**	**BrJ55-BrJ55**	Same forcing
	(3,9)	0.75	0.69	0.73	
	(9,15)	0.84	0.81	0.79	
	(3,15)	0.61	0.51	0.54	
		**BrE5-BrM2**	**BrE5-BrJ55**	**BrM2-BrJ55**	Cross-pairs
	3	0.80	0.85	0.86	
	9	0.74	0.81	0.82	
	15	0.76	0.81	0.82	
		**NOAA-BrE5**	**NOAA-BrM2**	**NOAA-BrJ55**	Rutgers
	3	0.47	0.53	0.50	
	9	0.42	0.49	0.46	
	15	0.34	0.41	0.36	

Table S1

Correlation values (tau) from Kendall-tau test. Test applied to detrended time series of October SCE. For the "same forcing" comparison, the two thresholds being compared are listed in the left column. For the two other comparisons, the column heading indicates the datasets being compared while the common threshold, applied only to B-TIM reconstructions, is shown in the left column.

	All B-TIM	B-TIM and NOAA
**High snow years**	1984, 1992, 1993, 1996, 2002, 2014, 2015, 2016	1984, 1993, 1996, 2002, 2014, 2016
**Low snow years**	1983, 1988, 1994, 2005, 2007, 2008, 2011	1988, 1994, 2005, 2007, 2008, 2011

Table S2

High and Low Snow Cover Years based on October SCE. After sorting by snow cover anomaly (i.e. snow-covered area minus linear trend), the ten highest snow years and ten lowest snow years are selected from each B-TIM dataset and from the NOAA CDR SCE. The years appearing in these lists for all three B-TIM datasets are listed in the left column, and those that appear for all three B-TIM datasets and in the list based on the NOAA CDR SCE are shown on the right. SCE is based on the 3mm threshold for the B-TIM datasets.

	RMSD (million km^2)		
**Method 1**			
**Month**	**BrE5**	**BrM2**	**BrJ55**
Sept	0.06	0.08	0.05
Oct	0.17	0.1	0.1
Nov	0.16	0.13	0.15
Dec	0.1	0.17	0.13
Jan	0.1	0.09	0.08
Feb	0.06	0.05	0.04
March	0.13	0.09	0.26
**Method 2**			
**Month**	**BrE5**	**BrM2**	**BrJ55**
Sept	0.82	0.77	1.01
Oct	0.64	0.56	0.72
Nov	1.13	1.21	1.05
Dec	0.83	1.27	1.13
Jan	0.67	0.97	0.81
Feb	0.57	0.64	0.83
March	0.53	0.51	0.72

Table S3
RMSD calculated between each dataset and NOAA CDR when optimal thresholds are chosen by each method. Each RMSD is calculated using 40 pairs of SCE values (40-90°N).

Month	Trends (million km^2/dec)
Sept	-0.07 ± 0.27
Oct	**-0.55 ± 0.49**
Nov	**-1.25 ±0.66**
Dec	**-0.71 ±0.44**
Jan	-0.26 ±0.40
Feb	-0.09±0.40
March	-0.06 ± 0.45

Table S4
JAXA JASMES monthly snow trends calculated for 40-90°N land. Bold indicates significance at the 95% confidence level.

REFERENCES AND NOTES

1. N. T. Boelman, G. E. Liston, E. Gurarie, A. J. H. Meddens, P. J. Mahoney, P. B. Kirchner, G. Bohrer, T. J. Brinkman, C. L. Cosgrove, J. U. H. Eitel, M. Hebblewhite, J. S. Kimball, S. LaPoint, A. W. Nolin, S. H. Pedersen, L. R. Prugh, A. K. Reinking, L. A. Vierling, Integrating snow science and wildlife ecology in Arctic-boreal North America. *Environ. Res. Lett.* **14**, 010401 (2019).

2. E. O. Aikens, M. J. Kauffman, J. A. Merkle, S. P. H. Dwinnell, G. L. Fralick, K. L. Monteith, The greenscape shapes surfing of resource waves in a large migratory herbivore. *Ecol. Lett.* **20**, 741–750 (2017).

3. J. Pulliainen, M. Aurela, T. Laurila, T. Aalto, M. Takala, M. Salminen, M. Kulmala, A. Barr, M. Heimann, A. Lindroth, A. Laaksonen, C. Derksen, A. Mäkelä, T. Markkanen, J. Lemmetyinen, J. Susiluoto, S. Dengel, I. Mammarella, J.-P. Tuovinen, T. Vesala, Early snowmelt significantly enhances boreal springtime carbon uptake. *Proc. Natl. Acad. Sci. U.S.A.* **114**, 11081–11086 (2017).

4. C. Mätzler, Passive microwave signatures of landscapes in winter. *Meteorol. Atmos. Phys.* **54**, 241–260 (1994).

5. M. G. Flanner, K. M. Shell, M. Barlage, D. K. Perovich, M. A. Tschudi, Radiative forcing and albedo feedback from the Northern Hemisphere cryosphere between 1979 and 2008. *Nat. Geosci.* **4**, 151–155 (2011).

6. D. J. Leathers, B. L. Luff, Characteristics of snow cover duration across the northeast United States of America. *Int. J. Climatol.* **17**, 1535–1547 (1997).

7. D. R. Gergel, B. Nijssen, J. T. Abatzoglou, D. P. Lettenmaier, M. R. Stumbaugh, Effects of climate change on snowpack and fire potential in the western USA. *Clim. Change* **141**, 287–299 (2017).

8. R. Petersky, A. Harpold, Now you see it, now you don’t: A case study of ephemeral snowpacks and soil moisture response in the Great Basin, USA. *Hydrol. Earth Syst. Sci.* **22**, 4891–4906 (2018).

9. T. P. Barnett, J. C. Adam, D. P. Lettenmaier, Potential impacts of a warming climate on water availability in snow-dominated regions. *Nature* **438**, 303–309 (2005).

10. A. R. Gottlieb, J. S. Mankin, Evidence of human influence on Northern Hemisphere snow loss. *Nature* **625**, 293–300 (2024).

11. World Meteorological Organization, “The 2022 GCOS Implementation Plan” [GCOS-244, World Meteorological Organization (WMO), 2022]; https://library.wmo.int/records/item/58104-the-2022-gcos-implementation-plan-gcos-244.

12. C. Derksen, L. Mudryk, Assessment of Arctic seasonal snow cover rates of change. *Cryosphere* **17**, 1431–1443 (2023).

13. D. E. Rupp, P. W. Mote, N. L. Bindoff, P. A. Stott, D. A. Robinson, Detection and attribution of observed changes in Northern Hemisphere spring snow cover. *J. Climate* **26**, 6904–6914 (2013).

14. Intergovernmental Panel On Climate Change (Ipcc), *The Ocean and Cryosphere in a Changing Climate: Special Report of the Intergovernmental Panel on Climate Change* (Cambridge Univ. Press, ed. 1, 2022; https://cambridge.org/core/product/identifier/9781009157964/type/book).

15. K. J. Bormann, R. D. Brown, C. Derksen, T. H. Painter, Estimating snow-cover trends from space. *Nat. Clim. Change* **8**, 924–928 (2018).

16. T. W. Estilow, A. H. Young, D. A. Robinson, A long-term Northern Hemisphere snow cover extent data record for climate studies and monitoring. *Earth Syst. Sci. Data* **7**, 137–142 (2015).

17. S. R. Helfrich, D. McNamara, B. H. Ramsay, T. Baldwin, T. Kasheta, Enhancements to, and forthcoming developments in the Interactive Multisensor Snow and Ice Mapping System (IMS). *Hydrol. Process.* **21**, 1576–1586 (2007).

18. M. J. Brodzik, R. Armstrong, Northern Hemisphere EASE-Grid 2.0 Weekly Snow Cover and Sea Ice Extent, Version 4, NASA National Snow and Ice Data Center Distributed Active Archive Center (2013); https://nsidc.org/data/nsidc-0046/versions/4.

19. R. D. Brown, C. Derksen, Is Eurasian October snow cover extent increasing? *Environ. Res. Lett.* **8**, 024006 (2013).

20. M. Hori, K. Sugiura, K. Kobayashi, T. Aoki, T. Tanikawa, K. Kuchiki, M. Niwano, H. Enomoto, A 38-year (1978–2015) Northern Hemisphere daily snow cover extent product derived using consistent objective criteria from satellite-borne optical sensors. *Remote Sens. Environ.* **191**, 402–418 (2017).

21. R. D. Brown, D. A. Robinson, Northern Hemisphere spring snow cover variability and change over 1922–2010 including an assessment of uncertainty. *Cryosphere* **5**, 219–229 (2011).

22. R. Urraca, N. Gobron, Temporal stability of long-term satellite and reanalysis products to monitor snow cover trends. *Cryosphere* **17**, 1023–1052 (2023).

23. L. Mudryk, M. Santolaria-Otín, G. Krinner, M. Ménégoz, C. Derksen, C. Brutel-Vuilmet, M. Brady, R. Essery, Historical Northern Hemisphere snow cover trends and projected changes in the CMIP6 multi-model ensemble. *Cryosphere* **14**, 2495–2514 (2020).

24. V. Eyring, S. Bony, G. A. Meehl, C. A. Senior, B. Stevens, R. J. Stouffer, K. E. Taylor, Overview of the Coupled Model Intercomparison Project Phase 6 (CMIP6) experimental design and organization. *Geosci. Model Dev.* **9**, 1937–1958 (2016).

25. X. Zhu, S.-Y. Lee, X. Wen, Z. Wei, Z. Ji, Z. Zheng, W. Dong, Historical evolution and future trend of Northern Hemisphere snow cover in CMIP5 and CMIP6 models. *Environ. Res. Lett.* **16**, 065013 (2021).

26. L. R. Mudryk, P. J. Kushner, C. Derksen, C. Thackeray, Snow cover response to temperature in observational and climate model ensembles. *Geophys. Res. Lett.* **44**, 919–926 (2017).

27. C. Mortimer, L. Mudryk, C. Derksen, K. Luojus, R. Brown, R. Kelly, M. Tedesco, Evaluation of long-term Northern Hemisphere snow water equivalent products. *Cryosphere* **14**, 1579–1594 (2020).

28. R. S. Kim, S. Kumar, C. Vuyovich, P. Houser, J. Lundquist, L. Mudryk, M. Durand, A. Barros, E. J. Kim, B. A. Forman, E. D. Gutmann, M. L. Wrzesien, C. Garnaud, M. Sandells, H.-P. Marshall, N. Cristea, J. M. Pflug, J. Johnston, Y. Cao, D. Mocko, S. Wang, Snow Ensemble Uncertainty Project (SEUP): Quantification of snow water equivalent uncertainty across North America via ensemble land surface modeling. *Cryosphere* **15**, 771–791 (2021).

29. E. Cho, C. M. Vuyovich, S. V. Kumar, M. L. Wrzesien, R. S. Kim, J. M. Jacobs, Precipitation biases and snow physics limitations drive the uncertainties in macroscale modeled snow water equivalent. *Hydrol. Earth Syst. Sci.* **26**, 5721–5735 (2022).

30. R. Brown, C. Derksen, L. Wang, A multi-data set analysis of variability and change in Arctic spring snow cover extent, 1967–2008. *J. Geophys. Res. Atmos.* **115**, D16111 (2010).

31. A. Elias Chereque, P. J. Kushner, L. Mudryk, C. Derksen, C. Mortimer, A simple snow temperature index model exposes discrepancies between reanalysis snow water equivalent products. *Cryosphere* **18**, 4955–4969 (2024).

32. T. Tian, S. Yang, J. L. Høyer, P. Nielsen-Englyst, S. Singha, Cooler Arctic surface temperatures simulated by climate models are closer to satellite-based data than the ERA5 reanalysis. *Commun. Earth Environ.* **5**, 111 (2024).

33. R. H. Reichle, Q. Liu, R. D. Koster, C. S. Draper, S. P. P. Mahanama, G. S. Partyka, Land surface precipitation in MERRA-2. *J. Climate* **30**, 1643–1664 (2017).

34. C. Mortimer, L. Mudryk, E. Cho, C. Derksen, M. Brady, C. Vuyovich, Use of multiple reference data sources to cross-validate gridded snow water equivalent products over North America. *Cryosphere* **18**, 5619–5639 (2024).

35. L. Mudryk, C. Mortimer, C. Derksen, A. Elias Chereque, P. Kushner, Benchmarking of SWE products based on outcomes of the SnowPEx+ Intercomparison Project. [Preprint] (2024). https://doi.org/10.5194/egusphere-2023-3014.

36. A. Elias Chereque, B-TIM snow for MERRA2, version 1, Borealis (2024); https://doi.org/10.5683/SP3/C5I5HN.

37. A. Elias Chereque, B-TIM snow for ERA5, version 1, Borealis (2024); https://doi.org/10.5683/SP3/HHIRBU.

38. A. Elias Chereque, B-TIM snow for JRA55, version 1, Borealis (2024); https://doi.org/10.5683/SP3/X5QJ3P.

39. E. Dutra, G. Balsamo, P. Viterbo, P. M. A. Miranda, A. Beljaars, C. Schär, K. Elder, An improved snow ccheme for the ECMWF land surface model: Description and offline validation. *J. Hydrometeorol.* **11**, 899–916 (2010).

40. L. Mudryk, C. Mortimer, C. Derksen, A. Elias Chereque, P. Kushner, Benchmarking of snow water equivalent (SWE) products based on outcomes of the SnowPEx+ Intercomparison Project. *Cryosphere* **19**, 201–218 (2025).

41. R. D. Brown, B. Brasnett, D. Robinson, Gridded North American monthly snow depth and snow water equivalent for GCM evaluation. *Atmos. Ocean* **41**, 1–14 (2003).

42. H. Hersbach, B. Bell, P. Berrisford, S. Hirahara, A. Horányi, J. Muñoz-Sabater, J. Nicolas, C. Peubey, R. Radu, D. Schepers, A. Simmons, C. Soci, S. Abdalla, X. Abellan, G. Balsamo, P. Bechtold, G. Biavati, J. Bidlot, M. Bonavita, G. De Chiara, P. Dahlgren, D. Dee, M. Diamantakis, R. Dragani, J. Flemming, R. Forbes, M. Fuentes, A. Geer, L. Haimberger, S. Healy, R. J. Hogan, E. Hólm, M. Janisková, S. Keeley, P. Laloyaux, P. Lopez, C. Lupu, G. Radnoti, P. de Rosnay, I. Rozum, F. Vamborg, S. Villaume, J.-N. Thépaut, The ERA5 global reanalysis. *Q. J. Roy. Meteorol. Soc.* **146**, 1999–2049 (2020).

43. R. Gelaro, W. McCarty, M. J. Suárez, R. Todling, A. Molod, L. Takacs, C. A. Randles, A. Darmenov, M. G. Bosilovich, R. Reichle, K. Wargan, L. Coy, R. Cullather, C. Draper, S.

Akella, V. Buchard, A. Conaty, A. M. da Silva, W. Gu, G.-K. Kim, R. Koster, R. Lucchesi, D. Merkova, J. E. Nielsen, G. Partyka, S. Pawson, W. Putman, M. Rienecker, S. D. Schubert, M. Sienkiewicz, B. Zhao, The modern-era retrospective analysis for research and applications, wersion 2 (MERRA-2). *J. Climate* **30**, 5419–5454 (2017).

44. S. Kobayashi, Y. Ota, Y. Harada, A. Ebita, M. Moriya, H. Onoda, K. Onogi, H. Kamahori, C. Kobayashi, H. Endo, K. Miyaoka, K. Takahashi, The JRA-55 reanalysis: General specifications and basic characteristics. *J. Meteorol. Soc. Jpn.* **93**, 5–48 (2015).

45. D. A. Robinson, A. Frei, Seasonal variability of Northern Hemisphere snow extent using visible satellite data. *Prof. Geogr.* **52**, 307–315 (2000).

46. Y.-K. Lee, C. Kongoli, J. Key, An in-depth evaluation of heritage algorithms for snow cover and snow depth using AMSR-E and AMSR2 measurements. *J. Atmos. Oceanic Tech.* **32**, 2319–2336 (2015).

47. M. G. Kendall, A new measure of rank correlation. *Biometrika* **30**, 81–93 (1938).
